# Willingness to pay for community based health insurance scheme and factors associated with it among households in rural community of South West Shoa Zone, Ethiopia

**DOI:** 10.1186/s12913-022-08086-z

**Published:** 2022-06-02

**Authors:** Mebratu Negera, Desu Abdisa

**Affiliations:** Department of Economics, Ambo University Woliso Campus, P.O.Box 217, Woliso, Ethiopia

**Keywords:** Community-based health insurance, Willingness to pay, Health care service, Contingent valuation, Ethiopia

## Abstract

**Background:**

Out-of-pocket payments are the major significant barrier in achieving universal health coverage, particularly in developing countries' rural communities. In 2011, the Ethiopian government launched a pilot community-based health insurance (CBHI) scheme with the goal of increasing access to modern health care services and providing financial security to households in the informal sector and rural areas. The main objective of this study is to estimate willingness to pay (WTP) for CBHI scheme and factors that influence it among rural households in the South West Shoa Zone.

**Methods:**

A household-level cross-sectional study was conducted to examine the WTP for the CBHI scheme and factors associated with it in rural communities of South West Shoa Zone. The study used a sample of 400 rural households. Systematic random sampling was employed during household selection, and double-bounded contingent valuation method was used to estimate WTP for the CBHI scheme.

**Results:**

About 65 percent of the households are willing to pay for CBHI scheme. Moreover, the study found that the households were willing to pay 255.55 Birr per year on average. The result of the study also revealed that amount of bid, household income, family size, household head's education, household health status, membership to community-based health insurance scheme, membership in any association, and awareness about the scheme are factors that are significantly associated with WTP for the scheme.

**Conclusions:**

Households are willing to pay a higher price than the policy price. Therefore, setting a new premium that reflects households' willingness to pay is highly valuable to policymakers. Social capital and awareness about CBHI scheme play an important role in influencing WTP. Thus, the study suggests that local communities need to strengthen their social cohesion and solidarity. It is also necessary to create awareness about the CBHI’s benefits.

**Supplementary Information:**

The online version contains supplementary material available at 10.1186/s12913-022-08086-z.

## Introduction

Achieving good health and well-being is one of the essentials for achieving Sustainable Development Goals [1]. As a result, developing countries have increased their efforts to achieve universal health coverage [2, 3]. The universal health coverage is defined as “everyone, regardless of their ability to pay, should be able to get the services they need without financial hardship” [4].

Health insurance is one of the options for achieving universal health coverage. It is a legal arrangement that protects insured individuals against the costs of medical services covered by the health insurance plan. Health insurance works best when the risk pools are large and the healthy can subsidize the sick [5]. There are four types of health insurance, depending on financial sources and characteristics: national health insurance, social health insurance, private insurance, and community-based health insurance [5]. National health insurance is a sort of government-managed insurance that covers all citizens and is supported by general taxation. Mandatory earmarked payroll contributions are used to fund social health insurance, and contributions and specified medical benefits are directly linked. Private voluntary insurance is commercial insurance administered by private for-profit companies, with premiums based on the purchaser's risk rather than his or her ability to pay.

CBHI is a non-profit organization founded on the principles of social solidarity and designed to safeguard households in the informal urban sector and rural areas from the catastrophic effects of out of -pocket medical expenses [6–9]. The ultimate purpose of CBHI is to achieve health system goals such as improving health status, health equity, health system responsiveness, and fairness in financial contribution [10]. Simplicity, accessibility, self-management, and complementing the public effort are all typical characteristics of CBHI schemes [8].

The Ethiopian health care system suffers from high reliance on out-of- pocket payments [11]. In Ethiopia, the bulk of healthcare spending (i.e., about 53%) came from household out-of-pocket payments, and 40% was contributed by the federal and regional governments [12]. Out-of-pocket payment has catastrophic effects, especially for households who mainly engage in agriculture and informal economies, which are characterized by less job security, lower incomes and absence of access to a range of social benefits [13, 14]. In 2011, the Ethiopian government launched a pilot community-based health insurance (CBHI) scheme with the goal of increasing access to modern health care services and providing financial security to households in the informal sector and rural areas [15, 16]. CBHI scheme is the best policy instrument to finance health care which can mitigate catastrophic health care expenditure, and can improve access to health care services in rural communities of Ethiopia [17, 18]. However, developing CBHI scheme, from a policy perspective, is relevant only if there is willingness to pay (WTP) for it [19].

In Ethiopia, only few studies have been done on WTP for community-based health care prepayment scheme among rural households of Ethiopia [20–22]. The study by [20] tried to estimate how much a household is willing to pay for CBHI scheme. However, they employed linear regression equation model in the presence of significant fraction of the observations with zero value for the dependent variable (WTP). In this case, standard Ordinary Least Square technique results in biased and inconsistent parameter estimates [23]. Even though the study conducted by [21] employed better regression model (i.e., interval regression model) as compared to the former one, the hypothetical market scenario choice set developed by them lacks clarity, and the findings may not apply in different contexts. Therefore, this study aims to fill these gaps through estimating WTP for CBHI scheme and identifying factors that associated with it among rural households in the South West Shoa Zone.

## Materials and methods

### Description of the study area

South West Shoa zone is one of the central zones of Oromia region which is located at a longitude of 37^0^ 05’ to 38^0^46’ from West to East and at a latitude of 8^0^16’ to 9^0^56’ from North to South. Like other zones of Oromia region, 90% of people of the zone live in rural areas, and they depend mainly on agriculture as their livelihood. Agriculture in the zone contributes enormous benefits to the people of the zone in particular and to the Ethiopian people in general. It is a source of food, source of income, raw-materials for agro-industries, and provides employment opportunity for rural households and their family members. The zone has abundant natural resources, especially watersheds and water resources. There are big rivers, streams, lakes and underground waters resulting in high potential irrigation in the zone. Even though it is traditional, irrigation is highly practiced in South West Shoa Zone.^1^

### Sample size and sampling technique

The target population of this study is rural households living in South West Shoa zone. This study covered all woredas^2^ because the researchers expected too much variation in WTP among households. This study used a simplified formula provided by Yamane (1967) to determine the sample size at 95% confidence level and 5% degree of variability (Israel, 2012). In addition, 5% level of precision was used in order to get the sample size which represents a true population. The sample size determination formula provided by Yamane (1967) is as follows [24].

$$\mathbf{n}=\frac{\mathbf{N}}{1+\mathbf{N}({\mathbf{e})}^{2}}$$

where $\mathrm{n}$ is the sample size, $\mathrm{N}$ is total number of farm households in South West Shoa Zone and

$$\mathrm{e}$$

 is the level of precision. According to report obtained from South West Shoa Zone Bureau of Agriculture and Natural Resource, there are 151,315 farm households in South West Shoa Zone in 2017. This study assumed that the level of precision is 5%.

$$\mathrm{n}=\frac{\mathrm{151,315}}{1 +\mathrm{ 151,315}{(0.05)}^{2}}\cong$$ 400 farm households.

We applied systematic random sampling during the household selection process. First, we decided the sampling interval by dividing the total households in the zone by determined sample size, which came to around 378. Next, we contacted kebele^3^ health extension workers to get a list of all households in each selected kebele. Finally, we employed systematic random sampling with the use of an interval 378 during household selection. Accordingly, one household was selected out of 378 households in each selected kebele, totaling 400 households.

### Sources of data and method of data collection

This research relies heavily on primary data. A questionnaire was used to collect primary data from a sample of farm households. Heads of farm households were interviewed by trained enumerators. This study's questionnaire consists of two sections. The first section of the questionnaire asks about farm households' demographic and socioeconomic characteristics, as well as their perceptions and awareness about CBHI scheme. The second section of the questionnaire covers the CBHI scheme's contingent valuation scenario as well as different WTP questions.

**Fig. 1:**
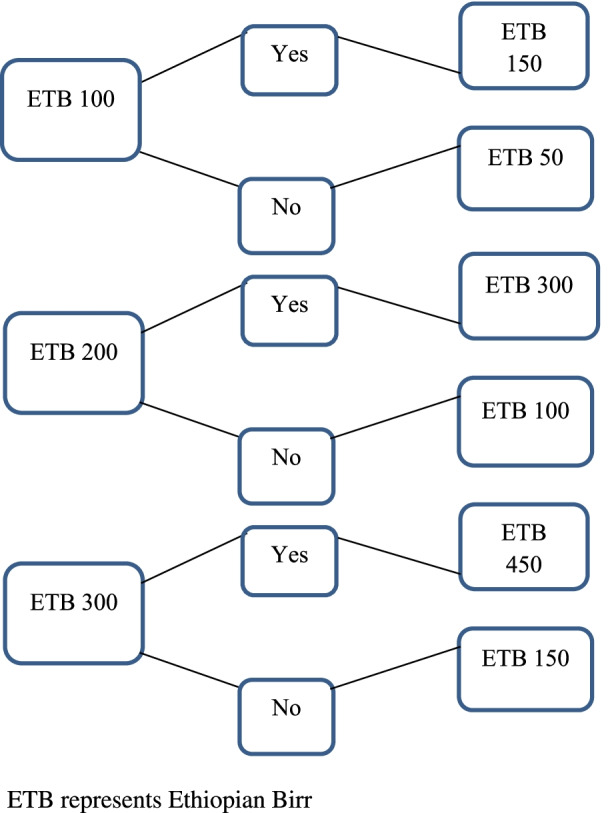
Double-bounded bidding technique

In the study, the double-bounded dichotomous choice format of CVM was used to elicit households' WTP for the CBHI scheme. Before conducting survey, focus group discussion (FGD) was conducted to generate initial bids. Figure 1 shows the double-bounded bidding technique used to elicit respondents' WTP in the study area. Three levels of the initial bid (Birr 100, Birr 200, and Birr 300) were established. The level of follow up bid depends on the response to the initial bid. If the respondent accepts the proposed initial bid, the follow-up bid becomes the initial bid plus half of the initial bid. On the other hand, if the initial bid is rejected, the follow-up bid becomes the initial bid minus half of the initial bid. Thus, Birr 50, Birr 100, and Birr 150 are the lower follow-up bids, whereas Birr 150, Birr 300, and Birr 450 are the higher follow-up bids.

Finally, we observed four joint responses when the sample households were asked the initial and follow-up bid questions: yes-yes, yes–no, no-yes, and no–no.

### Model specification

Seemingly unrelated bivariate probit model is used to estimate mean willingness to pay (MWTP) and identify factors that significantly influence rural households' WTP for the CBHI scheme. Let ${\mathrm{t}}^{1}$ denotes the initial bid and ${\mathrm{t}}^{2}$ the second bid. If ${\mathrm{WTP}}_{1\mathrm{i}}$ represents discrete response to the initial bid, and ${\mathrm{WTP}}_{2\mathrm{i}}$ is the discrete response to the follow-up following [25], the seemingly unrelated bivariate probit model can be specified as follows;

1 $${{\mathrm{WTP}}_{1\mathrm{i}}}^{*}={{{\upbeta }_{1}}^{\mathrm{^{\prime}}}\mathrm{X}}_{1\mathrm{i}}+{\upvarepsilon }_{1\mathrm{i}}$$

$${\mathrm{WTP}}_{1\mathrm{i}}=1$$ if $${{\mathrm{WTP}}_{1\mathrm{i}}}^{*}\ge {{\mathrm{t}}_{\mathrm{i}}}^{1}$$

$${\mathrm{WTP}}_{1\mathrm{i}}=0$$ if $${{\mathrm{WTP}}_{1\mathrm{i}}}^{*}<{{\mathrm{t}}_{\mathrm{i}}}^{1}$$

2 $${{\mathrm{WTP}}_{2\mathrm{i}}}^{*}={{{\upbeta }_{2}}^{\mathrm{^{\prime}}}\mathrm{X}}_{2\mathrm{i}}+{\upvarepsilon }_{2\mathrm{i}}$$

$${\mathrm{WTP}}_{2\mathrm{i}}=1$$ if $${{\mathrm{WTP}}_{2\mathrm{i}}}^{*}\ge {{\mathrm{t}}_{\mathrm{i}}}^{2}$$

$${\mathrm{WTP}}_{2\mathrm{i}}=0$$ if $${{\mathrm{WTP}}_{2\mathrm{i}}}^{*}<{{\mathrm{t}}_{\mathrm{i}}}^{2}$$

E $$\left({\upvarepsilon }_{1\mathrm{i}} |{\mathrm{X}}_{1\mathrm{i}},{\mathrm{X}}_{2\mathrm{i}} \right)=\mathrm{E}\left({\upvarepsilon }_{2\mathrm{i}} |{\mathrm{X}}_{1\mathrm{i}},{\mathrm{X}}_{2\mathrm{i}} \right)=0$$

$$\mathrm{Var}\left({\upvarepsilon }_{1\mathrm{i}} |{\mathrm{X}}_{1\mathrm{i}},{\mathrm{X}}_{2\mathrm{i}} \right)=\mathrm{Var}\left({\upvarepsilon }_{2\mathrm{i}} |{\mathrm{X}}_{1\mathrm{i}},{\mathrm{X}}_{2\mathrm{i}} \right)=1$$

$$\mathrm{Cov}\left({\upvarepsilon }_{1\mathrm{i}},{\upvarepsilon }_{2\mathrm{i}} |{\mathrm{X}}_{1\mathrm{i}},{\mathrm{X}}_{2\mathrm{i}} \right)=\uprho$$

This study used Krinsky and Robb approach to calculate the MWTP for the CBHI scheme since this approach takes the significance level into account when computing the MWTP.

## Results

### Socio-economic and demographic characteristics of households

Table 1 shows the descriptive statistics for the variables, with a focus on the mean, minimum, maximum, and standard deviation for continuous variables, and the percentage for categorical variables. The sample respondents' average household head age was 47 years old. On average, the heads of the sample households attended about 5 years in school, which is equivalent to a primary education in the current education system of Ethiopia. Furthermore, around 67 percent of the sample household heads were found to be literate. Male-headed and married households made up about 86 percent and 88 percent of the sample households, respectively. The sample respondents' average household size was around 6, implying that each household had roughly 6 people residing in it.

**Table 1:** Descriptive statistics of key variables

Continuous variables	Obs	Mean	Std. Dev	Min	Max
Household head age	400	47	11.12	19	81
Household head years of schooling	400	5	3.86	0	15
Family size	400	6	2.15	1	12
Dependency ratio	400	0.9	0.67	0	3
Monthly income	400	1,414.62	1,813.72	75	16,666.67
Monthly expenditure	400	967.01	1,036.98	100	10,000
Monthly saving	400	141.58	270.96	0	2,500
**Discrete variables**				**Obs**	**%**
Percentage of male-headed households				400	85.50
Percentage of married households				400	88.25
Percentage of literate headed households				400	67
Percentage of households who are member of CBHI				400	52
Percentage of households who are member of any association				400	54.50
Percentage of household heads who have responsibility in his/her community				400	34.75
Percentage of households faced sickness during the last three months				400	65
Percentage of household heads who practice saving				400	62.25
Percentage of households who are aware of CBHI scheme				400	89.50

When the survey was conducted, 65 percent of the sample households faced illness in the previous three months. Table 1 reveals that only 52 percent of the sampled households were members of the CBHI program, but about 90 percent of them were aware of its benefits.

### Descriptive analysis of response to bids

As presented in Table 2, the sample households' average open ended WTP is approximately Birr 223, with a zero minimum and Birr 500 maximum values. When compared to socio-economic variables like income, expenditure, and saving, the open ended WTP shows less variation across households.

**Table 2:** Descriptive statistics of open ended WTP and responses to bids

Continuous variable	Obs	Mean	Std. Dev	Min	Max
WTP	400	222.69	122.63	0	500
**Discrete variables**				**Obs**	**%**
Percentage of households who are willing to pay the initial bids				400	65.25
Percentage of households who are not willing to pay the initial bids				400	34.75
Percentage of households who are willing to pay the follow up bids				400	66
Percentage of households who are not willing to pay the follow up bids				400	34

Table 2 indicates that majority of the sample households are willing to pay the initial bids. Nearly 65 percent of those surveyed answered yes to the initial contingent valuation question and 66 percent of them answered yes to the follow up bid question. On the other hand, about 35 percent of households in the sample refuse to pay the first bid, and 34 percent refuse to pay the follow up bid question.

Together, the responses to the initial and follow-up bids form four possible joint responses. These responses are no–no, no-yes, yes–no and yes-yes. Therefore, households are assigned to one of these responses, and Table 3 summarizes the frequency distribution of the joint responses.

**Table 3:** Frequency distribution of joint responses

Response	Frequency	Percentage
No–No	33	8.25
No-Yes	106	26.5
Yes–No	103	25.75
Yes-Yes	158	39.5

For different groupings of sample households, the proportion of households with positive response to bids varies. Households can be classified into different groups based on a variety of socio-economic attributes. The chi-square test for discrete variable shows households that are literate, members of the CBHI scheme, members of any association, aware of the CBHI scheme, and have a position in the community have a higher proportion of households willing to pay the initial bid than their counterparts (see Table 4).

**Table 4:** Comparison of WTP between different groups

Variables	Groups	Percentage of households who are willing to pay the initial bid	Percentage difference	${{\varvec{\upchi}}}^{2}$
Sex of household head	Female	62.07	3.72	0.303
	Male	65.79		
Education	Illiterate	71.54	9.32	3.359*
	Literate	62.22		
Membership in CBHI	No	56.25	17.31	13.190***
	Yes	73.56		
Membership in any association	No	54.40	19.91	17.352***
	Yes	74.31		
Responsibility in a community	No	62.07	9.15	3.352*
	Yes	71.22		
Household health	Not healthy	66.54	3.68	0.544
	Healthy	62.86		
Practicing saving	No	63.58	2.69	0.300
	Yes	66.27		
Awareness about CBHI	No	35.71	33.01	18.055***
	Yes	68.72		

Note that *, ** and *** represent significance at 10%, 5% and 1%, respectively

**Fig. 2:**
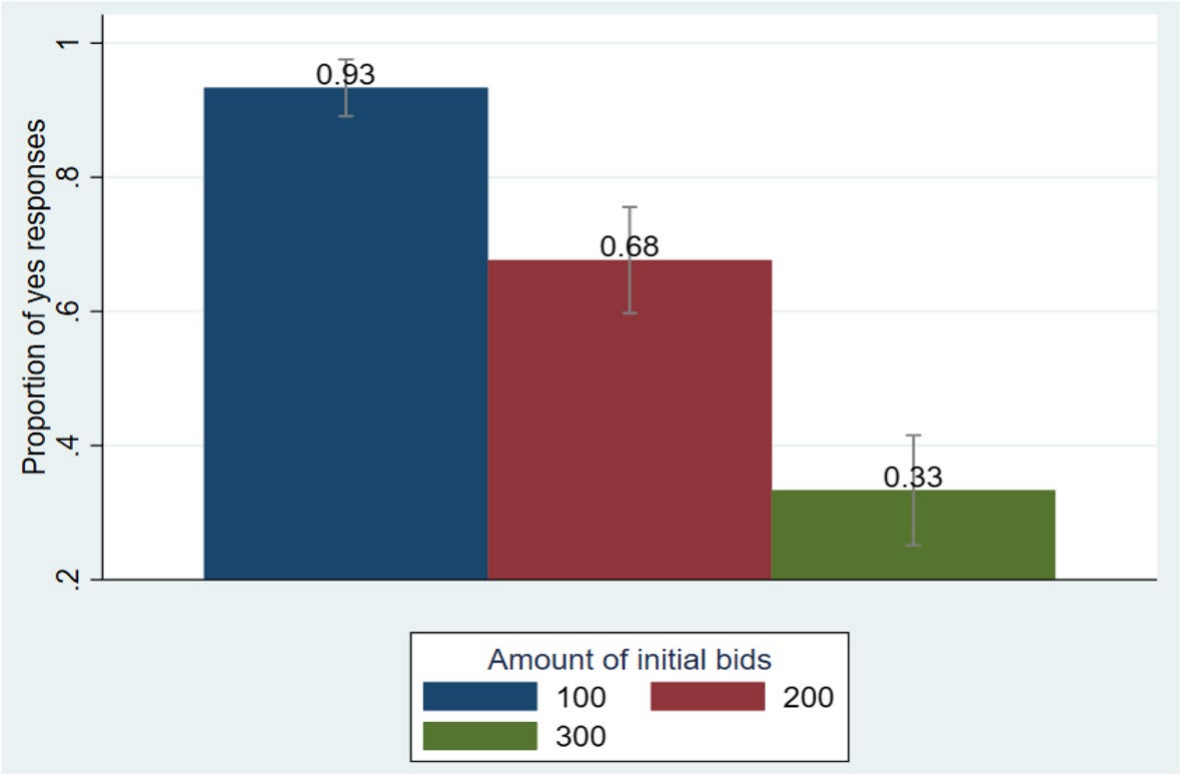
Confidence interval bar graph for response to initial bids

When employing contingent valuation data, it is critical to ensure that individuals are sensitive to the bid amount; that is, we expect that as the bid value increases, the proportion of respondents with positive response decreases. Figure 2 indicates that the proportion of households with positive response decreases as the bid amount increases. Besides, there is no region where the vertical lines in the bars overlap, implying that the proportion of positive response differs across the level of bids and the difference is significant.

### WTP for CBHI scheme and factors associated with it

Table 5 reports estimates of seemingly unrelated bivariate probit. The Wald test rejected the null hypothesis that all regression coefficients are simultaneously equal to zero [${\upchi }^{2}(26)=126.10$;$p>{\upchi }^{2}=0.000$]; implying that the model is of good fit. The correlation coefficient between response to the initial and follow up bids (Rho = -0.0094) is found to be statistically insignificant, as evidenced by the $P$ > ${\upchi }^{2}$ value of 0.961. This means that the initial bid response and the follow-up bid response are uncorrelated. As a result, estimation of seemingly unrelated bivariate probit model and equation-by-equation estimation of probit models provide the same result.

**Table 5:** Seemingly unrelated bivariate probit model regression result

Explanatory variables	Dependent variable		
	WTP_1_	WTP_2_	Marginal effect
Initial bid	-0.0105*** (0.0011)		-0.0024*** (0.0003)
Follow-up bid		-0.0035*** (0.0012)	-0.0009*** (0.0003)
Household income	0.0001* (0.0001)	0.0002** (0.0001)	0.0001*** (0.0000)
Health expenditure	0.0003 (0.0002)	0.0001 (0.0001)	0.0001** (0.0000)
Saving behavior	0.0827 (0.1688)	-0.1492 (0.1491)	-0.0198 (0.0548)
Sex of household head	0.2957 (0.2269)	0.4574** (0.2047)	0.1834** (0.0730)
Family size	-0.0728* (0.0414)	-0.0702** (0.0342)	-0.0348*** (0.0125)
Age of household head	0.0121 (0.0075)	0.0069 (0.0069)	0.0045* (0.0024)
Education level of household head	-0.0379* (0.0223)	-0.0090 (0.0199)	-0.0109 (0.0069)
Membership in CBHI	0.3856** (0.1629)	-0.1574 (0.1542)	0.0459 (0.0542)
Health status	0.3075* (0.1731)	0.0027 (0.1540)	0.0701 (0.0584)
Membership in any association	0.3942** (0.1740)	0.1150 (0.1503)	0.1187** (0.0550)
Responsibility in a community	0.1029 (0.1862)	0.1608 (0.1566)	0.0652 (0.0542)
Awareness about CBHI	1.0274*** (0.2728)	0.2837 (0.2264)	0.3041*** (0.0746)
Constant	0.9208 (0.5197)	0.4741 (0.4206)	
Log pseudo likelihood = -407.342 Number of observations = 400 Wald Chi^2^(26) = 126.10 Prob > Chi^2^ = 0.000		Athrho = -0.0094 (0.1922) Rho = -0.0094 (0.1922) Wald test of rho = 0: Chi^2^(1) = 0.0024 Prob > Chi^2^ = 0.961	

Note that values in the parentheses are robust standard errors. *, ** and *** represent significance at 10%, 5% and 1%, respectively

Following important empirical literatures, thirteen explanatory variables were used in seemingly unrelated bivariate probit regression to determine their effect on WTP for the CBHI scheme. Eight of these variables are statistically significant in influencing the response to the initial bid (see Table 5), which is the focus of our analysis. These variables are amount of bid, household income, family size, household head's education, household health status, membership to community-based health insurance scheme, membership in any association, and awareness about the scheme. While the amount of bid, family size, and household head's education level have a negative effect on the WTP for the CBHI scheme, household income, illness experience, CBHI membership, membership in any association, and awareness about the scheme have a positive effect.

We also calculated the WTP of households for the CBHI scheme following a seemingly unrelated bivariate probit regression. The MWTP for the CBHI scheme was calculated using the Krinsky and Robb method (see Table 6). To assess whether the MWTP estimates obtained by the Krinsky and Robb approach are adequate, the study compared them to the mean of open ended WTP.

**Table 6:** Estimated MWTP for CBHI scheme

Measure	WTP	LB	UB	ASL*	CI/MEAN
Mean/Median from equation(I)	255.55	239.47	273.96	0.0000	0.13
Mean/Median from equation (II)	336.35	278.67	552.16	0.0000	0.81

^*^indicates that Achieved Significance Level (ASL*) for testing H*_*0*_*: WTP* <  = *0 vs. H*_*1*_*: WTP* > *0*

*LB is Lower bound, UB is Upper bound*

The researcher must decide which MWTP to use if sets of MWTP are obtained in order to establish the value of the service in issue [26]. The MWTP computed from the first equation is chosen for different sensible reasons. First, the MWTP calculated from the first equation is close to the mean WTP of a contingent valuation question with an open-ended format (Birr 222.69). Second, the MWTP obtained from the first equation has a smaller variance as indicated by range. Third, the second equation employs household’s response to follow-up bid (as dependent variable), which is sensitive to bias because the household uses the first bid’s clue to make his WTP decision for the follow-up bid. Therefore, on average, the sample household in the study area is willing to pay Birr 255.55 per year (see Table 6). Therefore, multiplying the MWTP by the whole target population yields the welfare gain of Birr 38,668,548 per year.

## Discussion

The purpose of this study is to determine the WTP for CBHI and associated factors among households in South West Shoa Zone, Central Ethiopia. Around 65 percent of the households were willing to pay the first bid that was allocated to them at random. We found that households are willing to pay about 256 Birr per year. However, the Ethiopian government set the insurance premium at 240 Birr per household per year. This implies that the households are willing to pay a higher price than the policy price. This gives policymakers an important insight; they need to revise the current premium and set a new premium that reflects the households' willingness to pay.

In contingent valuation, the bid amount is expected to have a negative impact on the WTP. Table 5 (column 2) shows that when the bid amount rises, the probability of willing to pay the initial bid falls. This result is similar to that of [21] who found that as the price of health insurance increases, households are less willing to pay for CBHI scheme.

According to economic theory, the demand for necessities such as health insurance rises as the households' income rises. However, the change in demand in response to change in income is very small. The finding of this study suggests that when household income rises, the probability of willing to pay the initial bid rises as well. This result was iterated by [27–30], who indicated that demand for health care services, including health insurance, is income inelastic.

The study reveals that family size has a negative effect on the probability of WTP for the CBHI scheme, which is unexpected finding that contradicts many other studies. It means that households with a larger family size are less likely to be willing to pay than those with a smaller family size. One probable explanation is that households with a higher family size have higher non-health consumption expenditure like food, energy, and so on. In this situation, these groups of households spend less on modern health care services, such as health insurance.

A positive relationship between educational level and WTP was expected, but this study reveals that education has a significant negative effect on WTP. Despite the fact that this result contradicts many other findings, it is supported by [31].

Households in the study area, who are members of the CBHI scheme, are paying a premium decided by the government. Their WTP has not been taken into account by the government when determining this premium, indicating that the premium set by the government and households’ WTP for the CBH scheme are not equal. Since member households are aware of the scheme's benefits, it is believed that they would be more willing to pay than non-member households. This study's findings support this notion, indicating that membership in the CBHI scheme increases the probability of WTP for it.

The existence of health problems serves as the foundation for the provision of health care services and, as a result, health insurance. Because most people are risk averse, they are willing to join and pay for the CBHI scheme in order to avoid health-related risks. In particular, those who face illness frequently incur high out-of-pocket payments, hence they are more likely to join and pay for CBHI scheme. In this research, it also found that households who faced illness in the last three months are more willing to pay for the scheme than those who did not.

In rural areas membership in farmers' association builds social cohesion and solidarity, which are fundamental elements of social capital. The social capital facilitates collective action, which in turn, enhances WTP for CBHI scheme. As expected, regression result in this study indicates that WTP is positively influenced by social capital. Being a member of any association increases the likelihood of WTP for the CBHI scheme. This finding is consistent with many previous studies [32–34].

In the decision-making process, information, or knowledge, is very essential. In this study, WTP for the CBHI scheme shows a significant relationship with respondent’s awareness of the scheme. Households who are aware of the CBHI scheme are more willing to pay for it than those who are not. This might be due to the fact that households who are able to understand the catastrophic effects of out-of-pocket payments and benefits of joining the CBHI scheme are ready to join and pay for it. This finding is supported by research undertaken in Ethiopia [35], Nigeria [36, 37] and Cameron [19].

## Conclusion and policy implications

The results of the study have important implications. First, households are willing to pay a higher price than the policy price. This gives policymakers an important insight; they need to revise the current premium and set a new premium that reflects the households' willingness to pay. Second, social capital attributes such as membership in CBHI scheme and membership in any association play a vital role in WTP for CBHI scheme. Therefore, the local communities need to strengthen their social cohesion and solidarity. Third, especially in rural areas, it is critical to create awareness about the CBHI scheme's benefits. Finally, it is important to account the differences in WTP for CBHI schemes that appear across various groups when determining the amount of premium.

### Strengths and Limitations of the Study

The strength of this study is its large sample size and high response rate. Besides, it used double-bounded dichotomous choice format of CVM, which is more efficient than the single-bounded method. However, the study relies on cross-sectional data, which is ineffective in revealing unobserved factors influencing the WTP of the households. Moreover, the responses may be biased which can overestimate or underestimate the results of WTP.

## Supplementary Information

**Additional file 1.**

## Data Availability

The data will be available from Open Science Framework data repository, titled CBHI_data. xlsx with a link https://osf.io/edn5b

## Footnotes

1 The socio-economy profile of the zone is obtained from South West Shoa Zone Bureau of Agriculture and Natural Resource (2017).

2 Woreda is the third -level of the administrative division of Ethiopia – after zone and regional state.

3 Kebele is the smallest administrative unit in Ethiopia’s government structure – after woreda.
